# Use of the Internet by Patients Before and After Cardiac Surgery: An Interdisciplinary Telephone Survey

**DOI:** 10.2196/jmir.3.3.e27

**Published:** 2001-09-30

**Authors:** Monica Murero, Giuseppe D'Ancona, Hratch Karamanoukian

**Affiliations:** ^1^International Institute of InfonomicsHerleenNetherlands; ^2^Center for Less Invasive and Robotic Heart Surgery and Kaleida HealthBuffalo NYUSA

**Keywords:** Internet/statistics and numerical data, Questionnaires, Data Collection, Attitude to Computers, Coronary Artery Bypass, Patient Education, Readability

## Abstract

**Background:**

Little is known about to what extent patients who underwent medical treatment access the Internet and whether they benefit from consulting the Internet.

**Objective:**

To understand if cardiopathic patients use the Internet for health-related information and whether they find retrieved information understandable and useful.

**Methods:**

Telephone interviews, using a semi-structured questionnaire, were conducted with 82 patients who had undergone off-pump coronary-artery bypass grafting at the Center for Less Invasive and Robotic Heart Surgery in Buffalo, New York, USA. Study design was multidisciplinary, combining expertise of medical and communication science. Sources of medical information were identified (doctor, Internet, magazines, newspapers, television, radio, family members). Accessibility, quality, and readability of Internet medical information from the patients' point of view were investigated.

**Results:**

Out of 82 patients, 35 (35/82, 42.7%) were Internet users. Internet users had a significantly higher education level than Internet non-users (college education: 42.9% of users, 10.6% of non-users; P < .001). Among the Internet users, 18 (18/35, 51.4%) had used the Internet for retrieving medical information; 17 (17/35, 48.6%) had not. No statistically significant differences in demographic data were found when comparing these 2 sub-groups of patients. Family-members' involvement was high (15/18, 83.3%). Internet medical information was rated helpful in most cases; readability was acceptable for only 3 patients (3/18, 16.7%). To improve on-line medical information, all patients interviewed suggested sites designed by their physicians.

**Conclusions:**

Although 1 in 5 patients in our sample has used the Internet to retrieve medical information, the majority of them experiences difficulties comprehending the information retrieved. Health-care providers' should provide Internet medical information that is adequate for the non-medical public's needs.

## Introduction

It is crucial that patients have adequate medical information to educate, sensitize, and prepare themselves before and after undergoing medical procedures. Despite this, many fields of medicine remain unexplored by and unknown to the general public. Open-heart surgery remains one of the least-known fields, where myths and realities still coexist in the imaginations of patients. Coronary artery disease is one of the leading causes of mortality in the industrialized world; cardiopathic patients should be informed about indications, risks, and benefits of cardiac surgery, and its possible alternatives. Recent innovations in cardiac surgery (minimally-invasive and robotic procedures) have significantly decreased morbidity and mortality rates [1,2]. In many cases, coronary surgery can be performed without arresting the heart and without using the heart-lung machine [1]. Thanks to the contributions of a few pioneers, today open-heart surgery can be performed, in some instances, via small (5-cm long) incisions or even endoscopically, using robotic-assisted surgery [2].

Although clinicians and surgeons are aware of these new achievements, the majority of cardiopathic patients and their families, remain unaware of them. Adequate information on the Internet could increase patients' knowledge about these innovative surgical techniques and clarify their applications and limitations.

The introduction and popularization of the Internet is changing the way health-care providers and the general public search for and retrieve medical information. The Internet makes possible immediate access to and acquisition of encyclopedic information. In the field of cardiac surgery, for example, several authors have described and discussed the use of the Internet for educating cardiac surgery patients on-line [3,4,5,6]. However, little evidence is available about whether and to what extent this patient population actually uses the Internet. Although previous surveys have described the population of 'health-care seekers' that access and search Internet medical sites daily, they mostly do not distinguish between (a) patients that have already undergone or will undergo medical treatment and (b) the general public, including patients' family members, who may or may not have personal health-related problems [5]. Because most health resources available on the Internet are tailored for health-care providers, there is a question as to whether - even if patients access the Internet - patients understand and benefit from the information in these resources. We therefore focused our study on 2 central research questions: Is the Internet specifically used by cardiopathic patients to obtain health-related information? If this is the case, is on-line medical information understandable and useful for this non-medical public?

## Methods

Eighty-two patients that had previously undergone coronary artery bypass grafting (CABG) were interviewed by telephone during March and April 2001. The interdisciplinary approach followed in our research is built around a group of experts in the field of medical and communication science. Working together, the cardiac surgeon who performed the operations and a communication scientist specializing in Internet studies designed, tested, and conducted the interviews. To collect in-depth, quantitative and qualitative data, we used a semi-structured questionnaire [8]. During a pilot phase 2 alternative forms of the telephone script were tested [9]. The final semi-structured questionnaire used for data collection is shown in the Appendix. The communication scientist was present during the interviews.

All 82 patients who participated in the study were operated on with an innovative surgical technique consisting of construction of coronary anastomoses on the beating heart, avoiding use of the heart-lung machine (OPCAB: off-pump coronary artery bypass grafting) at the Center for Less Invasive and Robotic Heart Surgery in Buffalo, NY. Average time from surgery to questionnaire completion was 45 days.

In the first part of the interview, patients were asked about their general health condition. The surgeon provided professional advice while answering all health-related questions that the patients asked during the interview.

In the second part of the interview, questions were asked to investigate which sources (doctor, Internet, magazines, television, radio, family members) patients accessed to retrieve medical information. Specific questions were asked to investigate the use of the Internet before and after the operation. Internet users were asked to characterize accessibility, quality, and readability of Internet-mediated medical information. The overall benefits obtained by retrieving on-line medical information were investigated. All the Internet users were asked for suggestions for improving existing medical Internet sites.

### Statistical Analyses

Discrete and continuous variables were compared using the Pearson Chi-Square test and the Student t-test, respectively. Differences between variables were considered significant when the P value was less than .05.

## Results

The sample consisted of 82 patients. Demographics are provided in Table 1. Average age was 64.7 with standard deviation of 10.08 (range, 42-86) years, with a median of 67. Fifty patients (61%) were male and 32 (39%) were female. Twenty participants (24.4%) had college education, thirty-three (40.2%) high school education, and the remaining 29 (35.4%) had primary school or secondary school education. Out of 82 participants, 35 (42.7%) were Internet users and 47 (57.3%) had never used the Internet.

Education levels of Internet users and non-users were significantly different (chi ^2^= 29.78, with df = 2, P < .001). No other significant differences were observed in the demographics of the 2 groups (Table 1).

The most common means for acquiring medical information for patients (52/82, 63.4%) was via their family doctor, cardiologist, or cardiac surgeon (Table 2). The second most common means to acquire medical information was the Internet.

In the group of 35 Internet users, 2 subgroups were observed. Eighteen patients (51.4%) (subgroup A) had used the Internet to access medical information, and 17 (48.6%) (subgroup B) had never retrieved on-line medical information. No statistically-significant differences were observed when comparing the demographic characteristics (age, education, and gender) of these 2 subgroups (Table 3).

**Table 1:** Demographic Data for 82 Patients Interviewed

	**All Patients Interviewed (n=82)**	**Group of All Non-users of Internet (n=47)**	**Group of All Users of Internet (n=35)**	**Statistical Tests and P Value**
% of all patients interviewed	100%	57.3%	42.7%	
Average age, years Standard deviation	64.74 10.8 (range, 42-86)	66.40 8.88	62.51 11.24	P: NS* (t-test)
**Gender:** Male: % (number) Female: % (number)	61% (50) 39% (32)	63.8% (30) 36.2% (17)	57.1% (20) 42.9% (15)	χ ^2^= .377 df = 1 P: NS*
**Education:** College: % (number) High School: % (number) Primary School or Secondary School.: % (number)	24.4% (20) 40.2% (33) 35.4% (29)	10.6% (5) 29.8% (14) 59.6% (28)	42.9%(15) 54.3% (19) 2.9% (1)	^2^χ2 = 29.78 df = 2 P < .001

^*^ NS = not significant

**Table 2:** Sources of Medical Information for 82 Patients Interviewed

Source of Medical Information	Number of Patients Using the Source	% of Patients Interviewed Using the Source
Doctor	52	63.4
Internet	18	22.0
Magazines	16	19.5
TV	12	14.6
Family	5	6.1
None	4	4.8

**Table 3:** Demographic Data for 35 Patients with Internet Access

	**Subgroup A: Used Internet to Retrieve Health-related Information (n=18)**	**Subgroup B: Did Not Use Internet to Retrieve Health-related Information (n=17)**	**Statistical Tests and P Value**
% of patients with Internet access	51.4%	48.6%	
Average age, years Standard Deviation	60.21 2.37	64.4 9.67	P: NS* (t-test)
**Gender:** Male: % (number) Female: % (number)	61.1% (11) 38.9% (7)	47.1% (8/17) 52.9% (9/17)	χ^2^= .238 df = 2 P: NS*
**Education:** College: % (number) High School: % (number) Primary/Sec School: % (number)	50% (9) 50% (9) -	35.3% (6) 58.8% (10) 5.9% (1)	χ^2^= 1.625 df = 2 P: NS*

^*^ NS = not significant

### Details for subgroup A: patients who used the Internet to retrieve health-related information

Among the 18 patients who had obtained medical information from the Internet (subgroup A), the Internet was accessed before surgery in 9 cases (9/18, 50%), after surgery in 7 cases (7/18, 38.9%), and both before and after surgery in 2 cases (2/18, 11.1%).

Preoperative Internet use was mainly intended to retrieve general information about cardiovascular disease (7/18, 38.9%), general information about cardiac surgery (5/18, 27.7%), and specific information about innovative cardiac surgery procedures (eg, OPCAB) (3/18, 16.7%). Postoperative Internet use was more oriented towards general health and prevention issues (6/18, 33.3%) and towards information about cardiovascular drugs prescribed after the operation (3/18, 16.7%).

Fifty percent (9/18) of the patients involved at least 1 relative (other than a younger family member) in their search; in 3 cases (16.7%) a younger family member searched for the patient, and in 6 cases (33.3%) patients searched alone. Internet users in subgroup A, easily accessed the information using popular search-engines such as Yahoo! and AOL. In some cases (6/18, 33.3%) Internet users complained about 'overwhelming' information retrieved during the search.

Readability of the Internet-mediated medical information was found to be acceptable in only 3 cases (3/18, 16.7%). The majority (15/18, 83.3%) encountered difficulties in fully understanding the information. In spite of this readability barrier, the majority of patients in subgroup A (17/18, 94.4%) found the on-line medical information rather helpful to cope better with stress and anxiety during the preoperative and postoperative period.

The patients in subgroup A were asked for suggestions for improving the existing medical Internet sites. To increase the credibility of the medical information available on-line, all patients (18/18, 100%) indicated that they would benefit from access to Internet-sites developed by their immediate health-care provider (cardiologist or surgeon). Other suggestions are reported in Table 4. Fifteen patients agreed that medical information on the Internet should be reported using easier language in order to make it more understandable to the majority of the general public.

**Table 4:** Patients' Suggestions for Improving Existing Medical Internet Sites

**Patients' Suggestions**	**Number of Patients**	% of Patients*
Internet sites designed by the patient's doctor	18	100
Easier language	15	83.3
Patients' forum	3	16.7
Surgeons' results published	3	16.7
More links	2	11.1

^*^ Percentages are calculated on a total of 18 patients that did use the Internet to retrieve health-related information.

## Discussion

The Internet is a powerful tool in the hands of health-care providers and is also available to health-care users. However, many patients that could actually benefit from proper use of the Internet are unfamiliar with new technologies (eg, computer technologies) and, in the majority of cases, are not even on-line [10].

Fifty-seven percent of the interviewed patients have no Internet access. Based on age and gender no significant differences were observed comparing the groups of Internet users (35/82) and Internet non-users (47/82). However, statistically significant differences (P < .001) for levels of education were observed between the 2 groups. Well-educated patients use the Internet significantly more than poorly educated patients. Among Internet users, 42.9% (15/35) had a college education and more than half (54.3%, 19/35) had a high-school education. Among Internet non-users only 5 patients (10.6% 5/47) had graduated from college, while the majority had a primary school or secondary school education (59.6%, 28/47). This indicates that the more patients are educated the more they tend to be Internet users. This is consistent with the Stanford Institute for the Quantitative Study of Society study [10]. However, the level of education is not a predictor of the propensity to use the Internet for medical information retrieval: almost half (16/35, 45.7%) of the Internet users had either a high school or a college education and did not use the Internet for medical-information retrieval.

There were no significant differences in the demographic data (education, age, and gender) of the two subgroups (A and B) of Internet users. This brings up the question: why did about half (18) of the Internet users search for medical information and about half (17) did not? Behavioral and attitude differences in these 2 groups can be better explained by the social science literature [11,12]. Avoidance of using the Internet to retrieve information related to medical diseases could be interpreted, in part, as an attempt by patients to defensively prevent anxiety, since, being highly involved with a problem, the patients tend to avoid other sources of stimuli that could generate further dissonance, anxiety, and stress [13]. In this regard, direct consultation with the health-care provider may be more reassuring and less distressing. However, the Internet could be a potent means for retrieving information about conventional and innovative clinical and surgical treatments. In an original study entitled ROCEP (Rural On-line Cardiac Education Project), Scherrer et al [3] analyzed the effectiveness of the Internet, and other traditional methods of medical-education, in a group of patients waiting for cardiac surgery. The Internet-based method offered increased social support, decreased anxiety, and improved lifestyle - and facilitated positive attitudes towards the impending surgery. These findings are supported by a recent pilot study funded by NIH (National Institutes of Health) in which Leaffer et al [5] demonstrated that senior citizens can easily acquire computer and Internet skills to search for medical information on the Internet. Moreover, the elderly use this tool to assume an active role in their personal health care.

Berland et al [14] investigated accessibility, quality, and readability of Internet-based health information, and concluded that: access to health information is not efficient, coverage of key clinical information is poor and inconsistent, and high reading levels are required to understand Internet-based health information. Our findings support in part Berland et al's [14] conclusions from the patients point of view. Although the majority of patients could easily find general medical information, only a few patients (3/18, 16.7%) were actually able to retrieve specific information concerning innovative surgical treatments. Moreover, in most cases, the readability of information was poor.

Regarding the effects of using the Internet, patients reported an overall benefit from on-line information. However, significant concerns should be raised about the quality, credibility, and origin of health-related information available on the Internet. Although the Internet is a powerful tool for improving the health-care decision-making process, users should be aware of the potential for misinformation present in unprofessional Internet sites, and should always assess the source of information provided [15]. All patients agreed that, to increase the credibility of on-line medical information, access to Internet-sites developed by their immediate health-care-provider should be available. We believe that there is a growing need for objective, reproducible, widely accepted criteria that can form the basis for regulation of publication of medical material on the Internet. Internet users should be aware of the potential for on-line misinformation and should always be able to verify the source of information. Criteria for evaluating health information on the Internet have been presented in a policy paper supported by the Health Information Technology Institute [16].

### Limitations and Future Studies

This study represents the initial stage of an interdisciplinary project that includes ongoing research on larger cohorts of patients. The number of participants involved in the present study is limited because only a minority of cardiac-surgery candidates are currently treated with minimally-invasive surgical techniques (eg, OPCAB). In spite of the small size of the sample, demographic findings are consistent with the data published in the American Society of Thoracic Surgeons National Database [17].

The multidisciplinary approach of the present study has helped us to better understand the phenomenon under study and to address issues that stand in the nature of semi-structured questionnaire methodology. Under the supervision of the communication scientist, the interviewer (cardiac surgeon) avoided indirectly or directly suggesting or influencing patients' answers, thus preventing the 'Hawthorne effect' [18]. The "presence" of the interviewer (cardiac surgeon) may have increased cooperation rates and it also made it possible for respondents to get immediate clarification of health-related issues from their physician.

In conclusion, we believe that additional interdisciplinary studies should be encouraged to include not only cardiopathic patients, but also subjects affected with different pathologies, to investigate the relationship between type of disease and propensity towards Internet medical information retrieval.

## Abbreviations

CABG: Coronary Artery Bypass Grafting
OPCAB: Off-Pump Coronary Artery Bypass Grafting
ROCEP: Rural On-line Cardiac Education Project
NIH: National Institutes of Health

## Appendix 1

Semi-structured questionnaire used in the telephone interview

First part:

a) Surgeon's introduction and explanation of the reason for the interview

b) Check demographic data (name, age and education, gender)

c) Check actual health status of the patient

(-- Surgeon's feedback on post-surgery issues --)

Second part:*

d) Check all sources of information used when dealing with health-related issues

e) Check if Internet user or not-user.

f) Use of the Internet for medical-related topics (operation) (Yes/No)

- data access technique (web browsers, specific URLs, etc.)

- when the search was performed (before/after the operation)

- with whom (family-members' involvement in the search)

g) Quality of the data retrieved: (record patients' words) credible, clear (language)

h) Was the information retrieved helpful? (Yes/No)

Why? (check pros and cons of on-line medical information)

i) Suggestions for improving actual medical web sites'

* = Repeat key-questions (i.e., quality of information, pros and cons, problems encountered, benefits).
